# Psychological contributors to pain before, during, and after endodontic procedures: A scoping review

**DOI:** 10.1177/20494637251408718

**Published:** 2025-12-26

**Authors:** Atieh Sadr, Ali Gholamrezaei, Amy G. McNeilage, Cameron L. Randall, Flavia P. Kapos, Christopher C. Peck, Claire E. Ashton-James

**Affiliations:** 1 Sydney Dental School / Discipline Endodontics, 37580 The University of Sydney, NSW; 2 Pain Management Research Institute, Kolling Institute, Faculty of Medicine and Health, 4334 The University of Sydney; 3 Department of Oral Health Sciences, 114902 University of Washington School of Dentistry; 4 12277 Duke University School of Medicine; 5 Faculty of Dentistry, 37580 National University of Singapore, Singapore

**Keywords:** psychological, pain, endodontics, dentistry, dental management

## Abstract

**Background:**

Despite an increasingly biopsychosocial approach to pain management in healthcare, limited research exists on psychological features in dentistry including endodontic-related pain. This study aimed to identify the scope of evidence on the relationship of psychological variables to pain associated with dentistry specifically endodontics, as a first step towards addressing them for treatment.

**Methods:**

This scoping review was conducted according to the JBI Manual for Evidence Synthesis. Literature searches were performed in MEDLINE, EMBASE, PsycINFO, Web of Science, Scopus, Cochrane, and CINAHL, alongside grey literature sources, including ProQuest, ClinicalTrials.gov, and conference materials, reference lists, medRxiv pre-prints, EBSCO theses, and data from clinical trial registers such as ClinicalTrials.gov and Cochrane trials (via Ovid) (from inception to February 2025). Two independent reviewers screened records, and data extraction was cross-verified. The protocol was registered on Open Science Framework (DOI: 10.17605/OSF.IO/FSRJP).

**Results:**

Forty eight studies were included. Twelve broad psychological constructs were evaluated in relation to pre, during, and post-endodontic pain: pain expectancies, positive treatment expectancies, depression, anxiety, positive and negative mood (affect), beliefs about pain, desire for control of dental treatments, perceptions of dentists, somatic focus or awareness, pain coping strategies, personality, and psychiatric diagnoses. Pre-procedural pain was most frequently linked to anxiety. Procedural and post-procedural pain was consistently associated with anxiety, pain expectations, depression, and pain beliefs.

**Conclusion:**

A variety of psychological factors have been investigated in relation to endodontic pain at different time-frames. Whilst associations between endodontic pain and psychological constructs were found, further research is needed to evaluate the strength of these associations, and the scope of evidence for interventions designed to address these psychological contributors to pain in dental practice. Identifying psychological contributors to endodontic pain can enhance pain prediction, patient communication, and clinical care strategies.

## Introduction

Effective pain management is crucial in clinical endodontics,^1^ as research consistently links pain during dental procedures, including endodontics, to complications, persisting pain, and flare-ups.^2–4^ Current guidelines for dental pain management focus primarily on pharmacological approaches^5^: Non-steroidal anti-inflammatory drugs (NSAIDs) are recommended in combination with paracetamol as first-line treatments,^5–7^ and opioids like codeine and oxycodone as second-line treatments.^5–7^ Report of 5 to 49% of pain at different stages of endodontic procedures^2,8^ suggests that there is a need to improve our approach to pain management in dentistry.^4^

The fear-avoidance and cognitive-affective models of pain provide a conceptual framework for understanding how psychological constructs such as anxiety, depression, expectations, and feelings of helplessness shape the experience of endodontic pain. The fear-avoidance model suggests that these fear-related psychological constructs amplify pain by creating hypervigilance to threat and promoting avoidance behaviours.^9^ Also, the cognitive-affective model of pain suggests that beliefs about pain and anxiety could modulate pain experience because negative appraisals (e.g. expectation of uncontrollability) heighten attention to pain, increase threat perception, and activate emotional distress, which in turn intensify central nociceptive processing.^10^ Both these models support biopsychosocial approaches to pain management. Also medical literature provides substantial evidence that psychological factors such as preoperative anxiety, depression, and pain catastrophizing are associated with both greater acute procedural and post-operative pain and longer term chronic postsurgical pain.^11–13^

Recognizing pain as a biopsychosocial phenomenon is an important first step towards improving pain management in dentistry.^14^ A large body of research demonstrates the modulation of pain by a range of psychological factors.^15–17^ However, knowledge of psychological to endodontic pain remains limited.^18^ A systematic review found that psychological factors (e.g. Catastrophizing, beliefs, coping, and mood) are strong predictors in the transition from acute to chronic pain across many medical conditions.^19^

This paper focuses on the psychological aspects of pain and aims to address this gap in endodontics by using a systematic scoping review methodology to describe available research on psychological variables in endodontic-related pain. Specifically, the objectives of this review were to investigate psychological variables related to periprocedural (pre-procedural, procedural, and post-procedural) endodontic pain.

This initiative seeks to enhance the understanding of the psychological aspects in endodontic pain to guide pain management practices in this specialized field and assess the research body’s sufficiency for a potential systematic review and meta-analysis. Due to concerns about feasibility, the current review excludes an examination of social contributors to pain.

## Materials and methods

To uphold rigorous standards, our scoping review adhered to the Joanna Briggs Institute (JBI) Reviewer’s Manual^20^ and PRISMA Extension for Scoping Reviews (PRISMA-ScR) reporting guidelines.^21^ The research protocol was registered on Open Science Framework (DOI number: 10.17605/OSF.IO/FSRJP). The focus was on exploring the link between psychological factors and pain measures in English-language studies.

### Eligibility criteria, population, concepts, and context

The inclusion criteria encompassed studies involving patients undergoing endodontic procedures, including various study designs such as cohort, case-control, cross-sectional, clinical trials (interventional, observational), systematic reviews, and meta-analyses, with an evaluation of at least one psychological factor before, during, or after the endodontic procedure. Pain assessment for pre-procedural, procedural, or post-procedural, and reporting of the association between psychological factors and pain, was also required. Only studies published in English were considered. Exclusion criteria included case reports, case series, animal studies, letters, comments, editorials, and non-systematic reviews.

### Search strategy

The relevant keywords were selected by reviewing relevant articles and in consultation with a librarian. We conducted a pilot search on a random sample of 50 records retrieved from PubMed, and achieved 80% agreement in screening.^20^ The search strategy was then refined based on discrepancies identified during team discussions with experts in the field (Appendix 1).

We performed a search of multiple databases, including MEDLINE (via PubMed), EMBASE (via Ovid), PsycINFO (via Ovid), Web of Science (all databases), Scopus, Cochrane Database of Systematic Reviews (via Ovid), and CINAHL (via EBSCOhost). For the Grey Literature, we searched reference lists of the included studies as well as pre-prints available on medRxiv, theses via ProQuest Dissertations and Theses/EBSCO Open Dissertations, clinical trial registers such as ClinicalTrials.gov and the Cochrane Central Register of Controlled Trials (via Ovid), and conference materials including abstracts and proceedings via the Conference Proceedings Citation Index (via Web of Science) and Scopus (from inception to February 2025). To ensure the final search strategy adhered to the recommended guidelines, we employed the Peer Review of Electronic Search Strategies (PRESS) Checklist.^21^

### Study selection

We used EndNote and Covidence Systematic Review software^22^ for duplicate removal and screening. Literature selection followed predetermined eligibility criteria through a two-pass screening of titles, abstracts, and full texts, resolving disagreements through consensus by two-reviewer or third-reviewer intervention.

### Data extraction

There was no methodological quality or bias evaluation of studies included, in line with JBI^20^ guidelines for scoping reviews. A single reviewer extracted the data, with a second reviewer cross-verifying data in a random sample. Key information was recorded in a charting table for a descriptive summary aligned with the aims and objectives of the review.

## Results

**Figure 1.**
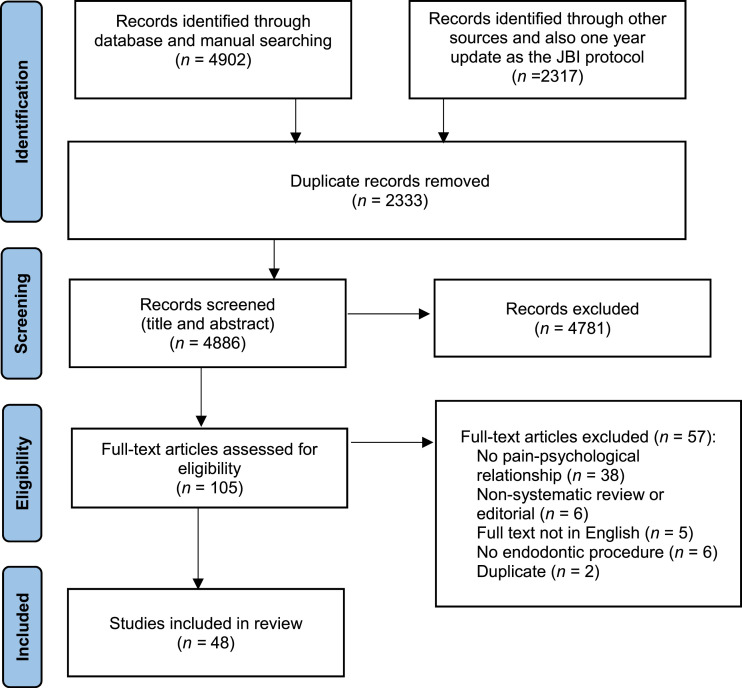
Flow chart of the studies selection process and screening.

The screened search ended with 48 articles for the final data extraction (Figure 1).

### Characteristics of the selected studies

The majority of the studies, comprising over two-thirds, are observational studies, predominantly prospective, with four retrospective studies and four cross-sectional studies, supplemented by four clinical trials. Fourteen studies^14^ were from North America (the United States and Canada), with 17 conducted in Europe (United Kingdom, Germany, France, Spain, Croatia, The Netherlands, Sweden, and Denmark), 9 conducted in Asia (Australia, China, India, Japan, Korea, Singapore, Taiwan), 4 conducted in the Middle East (Saudi Arabia, Israel, Iran), and 4 across multiple continents.

### Study participants

Studies included sample sizes ranging from 30 to 300 patients for observational studies and 1500 to 4800 for national surveys. Participant ages ranged from 6 to 86 years (mostly adults). Samples were 55% female on average, with 80% of patient cohorts coming from university educational dental clinics/hospitals and the remaining 20% from private surgeries.

### Pain measurement

**Figure 2.**
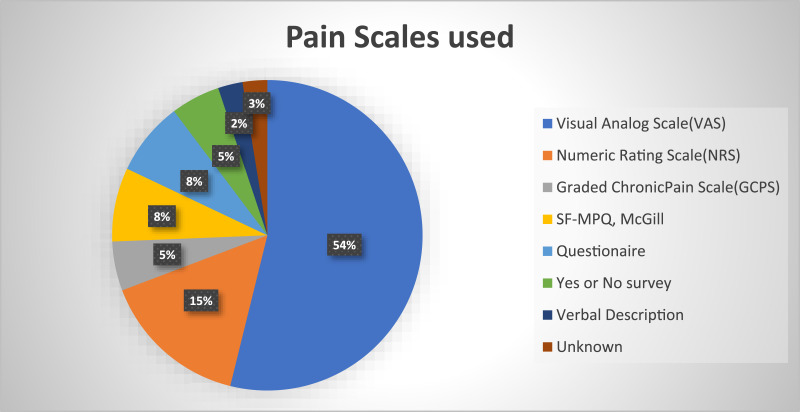
Details of scales used for pain measurement based on numbers.

Various self-reporting scales measured pain at different stages of the endodontic procedure (pre-procedural, procedural, and post-procedural). Pain intensity, frequently assessed by the Visual Analog Scale,^23^ was the most common outcome measured, as shown in Figure 2.

### Psychological constructs

Data were extracted from 48 studies (Table 1). Among the 48 studies identified, 11 investigated psychological variables associated with pre-procedural pain, 22 investigated psychological variables associated with procedural pain, and 24 focused on psychological variables associated with post-procedural pain (most studies investigated the predictors at one time frame of endodontic treatment, while nine studies investigated pain at two time frames). Psychological variables were organized under 12 higher-level psychological constructs, including ‘anticipated or expected pain’, ‘positive treatment expectation’, ‘depression’, ‘anxiety’ (anxiety encompasses various psychological variables such as state or trait anxiety, dental anxiety, fear, pain-related anxiety [like pain catastrophizing, fear of pain, psychological disability, social disability, and psychological discomfort], oral health–related anxiety, social functioning, and stress, these anxiety variables were grouped together given their strong correlation and frequent co-occurrence, to allow for easier synthesis), ‘beliefs about pain management’ (which encompasses variables such as the belief that stress exacerbates pain, expectations regarding the necessity of opioid medications for endodontic pain, and anticipations regarding pain relief after endodontic treatment), ‘desire for control of dental treatments’, ‘perceptions of dentists’ (this construct includes both perceptions of dentists and empathy of dentist), ‘somatic focus or awareness’, ‘positive and negative affect’ (often described as mood variable in the research), ‘pain coping strategies’, ‘personality’, and ‘psychiatric diagnosis’.

**Table 1.** Details of data extraction.

Author, year	Patients	Procedures	Psychological factors and measures	Pain measures	When the pain variable measured	Results
Ahmed et al., 2024	n = 100, F 55%	Endodontic treatment	Anxiety with MDAS and STAI	Pain measured with VAS	Post-procedural	Positive correlation
		Including RCT				
	Mean age 42					
Allebring et al., 1995	n = 48, F 90%	RCT; endodontics	Disease conviction measured with IBQ	Pain intensity measured with Likert scale	Post-procedural (time varied, months in most cases)	No correlation
	Age 30–81 y					
Alroomy et al., 2020	n = 95, F 56%	RCT	Anxiety measured with VAS	Pain intensity measured with ‘VAS-P’	Pre-procedural, before start of treatment in waiting room	Positive correlation
	Age 18–>60					
Applebaum and Maixner, 2009	n = 95, F 43%	RCT	Depression measured with BDI, somatization measured with PILL, anxiety measured with STAI	Pain intensity measured with Likert scale and Gracely box scale	Post-procedural, pain measured for 5 days consequently	Trait anxiety was correlated with post-operative pain
	Mean age 48 y					
	Age 30–81 y					
Arntz et al., 1990	n = 40, F 50%	Dental treatment including RCT	Dental anxiety with DAS, expected pain with VAS, and fear with VAS	Pain intensity measured with VAS	Procedural, immediately after about during treatment pain	Higher anxiety associated with overpredicted pain
	Mean age 34 y					
Attar and Baghdadi, 2015	n = 39, F 49%	Pulpotomy	Dental anxiety with MDAS	Pain measured using Wong-Baker FACES	Procedural, at different stages of RCT	No associations
	Mean age 6 y					
Baron et al., 1993	n = 188, F 60%	RCT	Control over dental treatment with IDCI	Pain intensity measured with VAS	Procedural, immediately after for during treatment pain	Negative correlation between desire for control or felt control with pain
	Mean age 41 y					
Brawley, 2019	n = 108, F 56%	Dental treatment including RCT	A self-report survey about drugs	Pain measured using DVPRS	Pre-procedural	Positive correlation
	Mean age 39 y					
Chandraweera et al., 2019	n = 69, F 61%	RCT	Expected pain measured with modified VAS	Pain measured with modified VAS	Procedural, after about during treatment pain	No correlation
	Mean age 49 y					
Cho et al., 2017	n = 44, F 64%	Apical surgery	Dental anxiety measured with MDAS	Pain measured with NRS	Procedural, straight after insertion and after injection asked about insertion and injection pain	Positive correlation
	Mean age 39 y					
Daline et al., 2020	n = 27, F 46%	RCT	Fear and optimism about the outcome with Likert scale	Pain measured with Likert scale	Post-procedural, after 6 months anytime	Positive correlation between fear and pain
	Mean age 46 y					
De Oliveira et al, 2024	n = 150, F 38 to 52%	RCT	Dental anxiety with MDAS	Pain measured with VAS and efficacy of injection	Pre-procedural and procedural	Positive correlation
Dou et al., 2018	n = 130, F 53%	Endodontics	Dental anxiety with MDAS and CARS	pain intensity measured with VAS	Pre and procedural (immediately after for during RCT pain)	Positive correlation for both
	Mean age 42 y					
Edwards et al., 2004	n = 46, F 67%	Emergency RCT	Catastrophizing with CSQ	Pain measured with NRS.	Pre-procedural	No correlation
	Mean age 36 y					
Edwards et al., 1999	n = 46, F 67%	Emergency RCT	Anxiety with STAI	Pain measured with NRS	Pre- and post-procedural	No correlation
			Mood with POMS-Bi and pain coping with CSQ			
	Mean age 36 y					
Eli et al., 1997	n = 92, F 38%	Dental treatment	State and trait anxiety with STAI, dental anxiety with DAS	Pain threshold, measured with VAS	Procedural	No correlation
		Including RCT				
	Mean age 28 y					
Farias et al., 2024	Four articles	Endodontics including RCT	Dental anxiety with MDAS, CDAS, SDAI, and VAS	Pain measured with VAS	Pre and procedural	Two studies found positive correlation
(Systematic review for pre and procedural pain and dental anxiety, all 4 studies included in this table)	471 total patients					
Gastmann et al., 2024	n = 60, all female (pregnant)	RCT	Dental anxiety with DAS	Pain measured with NRS	Pre- and post-procedural	For anxiety, no correlation
			Catastrophizing with PCS			Positive correlation for catastrophizing and post-procedural
Georgelin-Gurgel et al., 2009	n = 60, F 58%	RCT	Anxiety and discomfort and stress with VAS	Pain measured with VAS	Procedural, measured after about during procedural pain	No correlation
	Mean age 44 y					
Gochenour and McNeil, 2003	n = 63, F 56%	RCT	Fear with NRS	Pain measured with NRS	Procedural, at needle insertion, anaesthetic deposition (straight after), and access cavity preparation (not sure when exactly)	No correlation
	Mean age 43 y					
Khademi et al., 2021	n = 165, F 73%	Endodontics	Depression and anxiety with HADS, personality traits with NEO five-factor IS	Pain measured with VAS	Procedural,	Positive correlation with depression and neuroticism
					Pain was measured during procedure at needle insertion	
	Mean age 35 y					
Khoo et al., 2020	n = 150, F 45%	RCT or apical surgery	Psychological discomfort, psychological disability with OHIP-14	Pain measured with OHIP-14	Pre-procedural and post-procedural	Positive correlation with pre-procedural pain
	Mean age 52 y					
Klages et al., 2004	n = 97, F 57%	Tooth extraction or RCT	Dental anxiety with DAS, and expected pain with NRS	Affective and sensory pain measured with PES	Post-procedural	Positive correlation
	Mean age 39 y					
Law et al., 2014	n = 652, F 59%	RCT	Stress measured with Likert scale	Pain intensity measured with Likert scale	Post-procedural, measured 1 week after treatment	Positive correlation
	Mean age 48 y					
Laxmi et al., 2021	n = 46, F 43%	Endodontics	Dental anxiety with DAS	No details	Pre-procedural	Positive correlation
	Mean age 46 y					
LeClaire et al., 1988	n = 82, F 78%	RCT	Fear with Likert scale	Pain measured with Likert scale	Post-procedural, by a questionnaire	Positive correlation
	Mean age NR					
Lin et al., 2017	35 articles, F 55%	Dental treatments including RCT	Anxiety, fear, and expected pain measured in various ways across studies	Pain measured with various scales	Pre-, procedural, and post-procedural	Positive correlation of anxiety and pain
	Mean age 31 y					
Maggirias and Locker, 2002	n = 1422, F 58%	Dental treatment including RCT	Dental anxiety with DAS	Pain measured with Likert scale	Pre- and post-procedural, post at day 2, 4 and 7	Positive correlation
	Mean age 50					
Mannan and Greenspan, 2019	n = 39, F 59%	RCT	Anxiety/depression with HADS	Pain intensity measured with VAS	Pre- and post-procedural (immediately after treatment)	No correlation
	Mean age 44					
Mijailovic and Kolak, 2024	n = 140, F 42%	RCT	Depression, anxiety, and stress measured with (DASS)-21	Pain measured with VAS	Post-procedural	For anxiety, positive correlation
	Mean age 39 y					Stress and depression, no correlation
Miura et al., 2018	n = 383, F 85%	Dental treatment including RCT	Psychiatric diagnoses with ZSRDF	Pain measured with SF-MPQ	Post-procedural,	Positive correlation
					Post-emergency treatment, not exact time recorded	
	Mean age 54					
Mohorn et al., 1995	n = 30, F 53%	Emergency endodontics	Anxiety with state-trait STAI and CSQ and POMS	Pain measured with MPI	Pre- and post-procedural	Positive correlation between negative mood and pain
	Mean age NR					
Murillo-Benítez et al., 2020	n = 175, F 51%	RCT	Dental anxiety with S-DAI	Pain measured with VAS	Pre-procedural (immediately after treatment) and procedural	Positive correlation
	Age range, 18 to 68 y					
Nixdorf et al. (Nixdorf et al., 2016)	n = 651, F 59%	RCT	Fear of appointment with a Likert scale	pain intensity measured using GCPS	Pre- and post-procedural, pain measured at 6 months after treatment	Negative correlation of optimism about the procedure and anxiety
	Mean age 48					
Nogueira et al., 2024	n = 62	RCT	Psychological discomfort (stress, anxiety, and difficulty relaxing) was assessed using the OHIP-14	Pain measured with NRS	Procedural	Positive correlation
	Mean 34 y					
Peretz et al., 1998	n = 98, F 57%	RCT	Dental anxiety with DAS	Pain measured with a Likert scale	Pre-procedural (not exact timing)	No correlation
	Mean age 45					
Perković et al., 2014	n = 66, F 58%	Endodontics	Anxiety with CDAS, expected pain with Likert scale	Pain measured with VAS	Pre and procedural	No correlation for procedural and positive correlation for pre
	Mean age 34					
Philpott et al., 2019	n = 198, F NR	RCT or RCT retreatment	Pain catastrophizing with PCS	Pain measured using SF-MPQ	Post-procedural, pain measured 5 to 12 months	No correlation
	Mean age NR					
Pillai et al., 2020	n = 37, F 65%	Extraction or endodontics	Distress and somatic symptom severity and psychological discomfort with PHQ-4/PHQ-15), and OHIP-49 and PCS and BDI-II and STAI	Pain measured with MPQ) and alsoGCPS	Post-procedural	Positive correlation between the examined psychological predictors and pain
	Mean age 45					
Rahman et al., 2011	n = 337, F NR	Dental treatments including RCT	Anxiety and depression with HADS	Pain measured with VAS	Post-procedural, pain recorded in 20 days diary	Positive correlation
	Mean age NR					
Sorrell and McNeil, 2003	n = 104, F 58%	RCT	Fear with NRS, fear of pain with FPQ-III and DFS	Pain measured with NRS	Pre-, procedural, and post-procedural	No correlation
	Mean age 44					
Silva et al. 2023	n = from 58 to 100	RCT	Anxiety with CDAS	Pain measured with HF-VAS	Procedural	Positive correlation
			Anxiety related to injection with MDAS			
Thom et al. (Thom et al., 2000)	n = 50, F 52%	Extraction or endodontics	Dental anxiety with DAS and fear with DFS and STAI	Pain intensity measured with VAS	Post-procedural	No correlation
					Measured immediately after, 1 week and 1 month after	
	Mean age 30					
Vitali et al., 2024	n = 154, F 54.5%	RCT	Dental anxiety with DAS	Pain measured with	Post-procedural	All psychological predictors had positive correlation
			Dental fear with DFS	NRS		
	Mean age 33 y		Sense of coherence with SOC-13			
Watkins et al., 2002	n = 333, F 61%	Endodontics	Stress with STAI and IDCI	Pain measured with VAS	Procedural, measured immediately about procedural pain	Positive correlation
	Mean age 45					
Weitz et al., 2020	n = 531, F NR	RCT	Fear with Likert scale	Pain measured with Likert scale	Pre-procedural and procedural	Positive correlation
	Mean age 47					
Wu et al., 2022	n = 35, F 57%	Emergent endodontics	Anxiety with NRS	Pain measured with NRS	Pre- and post-procedural	Positive correlation
	Mean age 46					
Yang et al., 2016	n = 4866, F 56%	Dental treatments including RCT	Depression with Likert scale	Pain measured with VAS	The data is from a national survey; the exact time of measurement is not recorded	Positive correlation
	Mean age 45					

All scales abbreviations: Illness Behavioural Questionnaire (IBQ), Visual Analog Scale (VAS), Depression with Depression Index (BDI), Somatization with Pennebaker Inventory of Limbic Languidness (PILL), State-Trait Anxiety Inventory (STAI), Modified Dental Anxiety Scale (MDAS), Wong-Baker FACES, Iowa Dental control Index (IDCI), Defense and Veterans Pain Rating Scale (DVPRS), Numerical Rating Scale (NRS), Coping Strategies Questionnaire (CSQ), Thermal pain onset (TPO) and tolerance (TPT), Clinical Anxiety Rating Scale (CARS), the Profile of Mood States-Bipolar (POMS-Bi), Dental Anxiety Scale (DAS), Hospital Anxiety and Depression scale (HADS), the short form of NEO Five-Factor Inventory scale (NEO Five -Factor I), Thermal pain onset (TPO) and tolerance (TPT), Oral Health Impact profile 14 (OHIP-14), the Patient Health Questionnaire 4 and 15 (PHQ-4/PHQ-15, Corah’s Dental Anxiety Scale (CDAS), Pain Experience Scale (PES), Zung’s self-rating depression scale (ZSRDS), Multidimensional Pain Inventory (MPI), Graded Chronic Pain Scale (GCPS), the Oral Health Impact Profile 49 (OHIP-49), Pain Catastrophizing Scale (PCS), the Beck Depression Inventory-II (BDI-II), Fear of Pain Questionnaire-III (FPQ-III), Dental Fear Survey (DFS), Sense of coherence-13 (SOC-13).

Taken together, the evidence suggested that psychological constructs had influence on endodontic pain. Pre-procedural pain appears to have association with two constructs of anxiety and ‘beliefs about pain management’. Procedural pain showed the stronger associations with anticipatory cognitions such as expected pain, alongside anxiety, depression, and some other constructs mentioned. Post-procedural pain demonstrated the most consistent psychological influences, particularly anxiety, coping strategies, and desire for control, highlighting the role of affective and behavioural factors after treatment (Table 2). Appendix 1 shows the search codes used in different databases and Appendix 2 describes the scales used to measure each of the reported psychological variables associated with pre-procedural, procedural, and post-procedural pain.

**Table 2.** Summarized comparison of psychological constructs found associated with pre-procedural, procedural, and post-procedural pain.

Pre-procedural pain	Psychological construct	Subtype insights	Number of studies and Number of studies with significant correlation
	Anxiety	Various predictors: State anxiety (2 studies), dental anxiety (7 studies), oral health–related anxiety (1 study). Anxiety linked to fear of pain, procedural concerns, and psychological discomfort	10 studies
			6/10 studies showed correlation
	Beliefs about pain management	Expectations regarding opioid use and its necessity linked with pre-procedural pain anticipation	1 study
			1/1 study showed correlation
Procedural pain	Anxiety	Various predictors: Procedural pain linked to fear, dental phobia, and pre-existing anxiety. Studies used tools such as VAS and anxiety-specific questionnaires	18 studies
			9/18 studies showed correlation
	Depression	Indicators such as emotional states and psychological predispositions (e.g. lack of coping mechanisms) linked to pain intensity	2 studies
			2/2 studies showed correlation
	Expected pain	Predictors included prior experiences and procedural expectations. Tools: PES, NRS, VAS	5 studies
			4/5 studies showed correlation
	Positive treatment expectations	Linked to patient optimism regarding outcomes and reduced procedural distress. Measured via pre-treatment surveys	One study
			1/1 study showed correlation
	Personality	Neuroticism as a predictor. Tools included personality trait surveys	One study
			1/1 study showed correlation
Post-procedural pain	Anxiety	Subtypes: Dental, state, trait, and anticipatory anxiety. Predictors included fear of post-treatment pain and stress	18 studies
			14/18 studies showed correlation
	Depression	Linked to chronic pain and reduced coping ability. Tools: HADS, BDI-II	7 studies
			4/7 studies showed correlation
	Expected pain	Relief expectations often influenced by prior experiences and emergency treatments	One study
			0/1 study showed correlation
	Positive treatment expectations	Optimism about outcomes and reduced long-term pain reported	Two studies
			1/2 studies showed correlation
	Desire for control	Control over treatment linked to reduced anxiety and pain severity. Tools: ICDI	Two studies
			2/2 studies showed correlation
	Perceptions of dentists	Empathy and professionalism linked to lower pain perception	Two studies
			2/2 studies showed correlation
	Somatic focus or awareness	Chronic pain conditions linked to heightened somatic awareness. Mixed findings overall	Three studies
			1/3 studies showed correlation
	Psychiatric disease	Significant psychiatric predictors included melancholy and stress. Tools: National surveys	One study
			1/1 study showed correlation
	Pain coping strategies	Coping inefficacy (e.g. high avoidance) linked to increased pain severity. Tools: CSQ	Two studies
			2/2 studies showed correlation
	Beliefs about pain management	Stress as a pain exacerbator identified as a key predictor	One study
			1/1 study showed correlation
	Positive and negative affect	Mood predictors (positive or negative) showed minimal correlation with outcomes	Two studies
			0/2 studies showed correlation

### Psychological constructs associated with pre-procedural pain

Two psychological constructs – anxiety and beliefs about pain – were investigated in association with pre-procedural pain.^24–31^

#### Anxiety

Ten studies investigated various forms of anxiety in relation to pre-procedural pain. State anxiety was measured in two studies,^24,31^ while dental anxiety was measured in seven studies,^27–30,32,33^ and one study^26^ measured oral health–related anxiety. Six of the 10 studies reported a correlation between some form of anxiety and pre-procedural pain of endodontics.^24,26–28,31^

#### Beliefs about pain management

One study^25^ measured patient perceptions of pain relief and expectations of receiving an opioid medication together with pre-procedural pain. The study reported a positive relationship between the expectation of receiving an opioid medication and pre-procedural pain.

### Psychological constructs associated with procedural pain

Numerous studies investigated the association between psychological variables and procedural pain, with 20 studies identified in this review.^18,28,30,34–50^ The five psychological constructs investigated for procedural pain were anxiety, depression, expected pain, positive treatment expectations, and personality.

#### Anxiety

Eighteen studies investigated the association between various forms of anxiety and procedural pain^18,28,30,34–44,46,48–50^ with mixed results. Of these, 9 out of 18 studies reported a significant positive association between anxiety and procedural pain.

#### Depression

Two studies^42,45^ reported a positive correlation between depression and procedural pain.

#### Expected pain

Expected pain was evaluated in relation to procedural pain of endodontic treatment in five studies.^36,39,43,46,47^ Expected pain was measured using various tools including visual analog scales (VAS), numerical rating scales (NRS), and the Pain Expectation Scale (PES). Four of these studies^36,39,43,47^ showed a positive association between pain expectation and procedural pain.

#### Positive treatment expectations

Weitz et al.^48^ was the only study in this category that measured the expected outcome of root canal therapy for procedural pain, reporting a significant association between higher outcome expectancy and anaesthesia failure or procedural pain.

#### Personality

Personality was investigated by one study,^42^ which reported a positive correlation between neuroticism scores and pain experience during needle insertion for injection or procedural pain.

### Psychological constructs associated with post-procedural pain

The present review included 24 studies that investigated the relationship between 11 psychological variables and post-procedural pain, including anxiety, depression, ‘expected pain’, ‘positive treatment expectations’, ‘positive and negative effect’, ‘pain coping strategy’, ‘desire for control’, ‘perception of the dentist’, ‘somatic focus and awareness’, ‘psychiatric disease’, and ‘beliefs about pain’.^18,30,31,33,51–64^

#### Anxiety

Anxiety was investigated in relation to post-procedural pain in 18 studies,^18,26,30,31,33,43,52,53,56–65^ with 14 different anxiety measures used. Overall, 14 out of 18 studies reported a significant association between anxiety and post-procedural pain.

Three studies measured state and trait anxiety, one with a pain diary questionnaire and the other with NRS.^31,59,61^ Nine studies measured dental anxiety, using an 11-point NRS, Graded Chronic Pain Scale (GCPS), or another questionnaire (details below).^18,30,43,52,56,65^ Four studies measured pain catastrophizing using either the Pain Catastrophizing Scale (PCS) or the Pain Coping Scale (PCS).^33,53,57,58^ Two studies measured oral health–related anxiety, one with the Oral Health Impact Profile Questionnaire (OHIP-14), measuring psychological discomfort, psychological disability, and social disability,^26^ and the other with PHQ-4, PHQ-15, and OHIP-49, measuring discomfort, somatic symptom, and psychological disability.^58^ One study measured stress through a questionnaire in a national survey.^60^ Both state and trait anxiety were significantly correlated with post-procedural endodontic pain,^31,59^ with trait anxiety (HADS) identified as a significant predictor of pain in the first 5 days after endodontic treatment.^59^ Seven out of nine dental anxiety studies showed a positive correlation between post-procedural pain and anxiety, while two (NRS, GCPS) found no relationship.^18,56^ One study^30^ showed a significant correlation between pain and the Dental Fear Survey-Physiological Score (DFS), but had limitations of low sample size. Except for Pillpott et al.,^57^ two out of three studies investigating pain catastrophizing reported a significant correlation with post-procedural endodontic pain.

#### Depression

Seven studies evaluated the relationship between depression and post-procedural pain. Three studies^57,63,66^ showed no significant relationship between depression and post-procedural pain, and four studies^45,58,60^ reported a significant association between depression and post-procedural pain. Mannan and Greenspan^45^ and Rahman et al.,^59^ who used the Hospital Anxiety and Depression Scale (HADS) to measure depression, found depression increases post-procedural pain. Pillai et al.^58^ and Yang^60^ reported higher pain in their depressed groups, measures using the Beck Depression Inventory-II and a questionnaire, respectively, after root canal treatment or in patients with Post-Traumatic Trigeminal Neuralgia (PTTN), a chronic pain condition after dental treatment.

#### Expected pain

Only one study^31^ examined the correlation between expected pain relief and post-procedural pain after emergency endodontic treatment. There was no significant correlations between expected pain and post-procedural pain.^31^

#### Positive treatment expectations

Two studies examined positive treatment expectations in relation to post-procedural pain.^31,52^ Daline et al.^52^ assessed expected outcomes through a questionnaire in patients experiencing chronic pain after root canal therapy and reported no association between long-term persistent pain and patients’ optimism about the treatment outcome measured during the 6 months after treatment. However, Wu et al.^31^ evaluated expected pain relief in patients undergoing emergency endodontic treatment and found a positive correlation between expected pain relief and post-procedural pain reduction.

#### The desire for control over dental treatment

The desire for control over dental treatment was investigated in relation to post-procedural pain in two studies,^51,65^ both of which reported a significant correlation between this psychological construct and post-procedural pain using the Iowa Dental Control Index (ICDI).

Baron et al.^51^ assessed patients’ desire for control and felt control in the dental chair 30–40 min before the start of treatment. Participants were asked about the degree to which they wanted to have control over dental treatment and how much they felt they had control. The high desire for control or felt control was reported to be associated with lower pain and higher sensory focus than emotional focus measured with a questionnaire. In addition, Maggirias and Locker^65^ reported that pain was significantly higher in participants with higher scores on the ICDI Felt Control scale than in those who did not report experiencing pain.

#### Perceptions of dentists

Positive perceptions of the dentist was evaluated in relation to post-procedural pain in two studies^46,65^ which both reported negative correlations with pain. Maggirias and Locker^65^ assessed patients’ perceptions of the dentist and how dental care was delivered during endodontic treatments using a baseline question to assess whether patients had ever had a painful, frightening, or embarrassing experience with a dentist. They found a negative correlation between the high reported empathy of dentists and post-procedural pain onea week after treatment.

#### Somatic focus or awareness

Three studies reported mixed results regarding the relationship between somatic focus or awareness and post-procedural pain.^58,60,61^ Pillai et al.^58^ measured somatic symptom severity using the Patient Health Questionnaire 15 (PHQ-15) before various dental treatments, including endodontics, and reported that patients with painful post-traumatic trigeminal neuropathy (PTTN), which is a chronic pain condition, had higher scores for somatic symptoms than healthy controls. Conversely, Applebaum and Maixner^61^ measured somatization using the Pennebaker Inventory of Limbic Languidness (PILL) completed after root canal treatment (within 5 days after treatment) and found no significant association between post-treatment somatization and post-procedural pain.

#### Psychiatric disease

One study^60^ used a national survey to measure melancholy, consultation with a psychiatrist, and suicidal thoughts before various dental procedures, including root canal therapy, and found significant differences in melancholy, suicidal thoughts, or consultation with a psychiatrist between those with and without self-reported dental pain.^60^

#### Pain coping strategies

Two studies^54,55^ used the Coping Strategies Questionnaire (CSQ) to measure pain coping before emergency endodontic treatment and post-procedural pain (1-2 weeks after surgery). Both these studies reported that higher scores on CSQ subscales were significantly associated with higher post-procedural pain.^54,55^

#### Beliefs about pain management

Beliefs about pain were evaluated in only one study.^67^ The study administered a questionnaire to patients before root canal treatment to assess any relationship between the belief that ‘stress makes the pain worse’ and severe post-procedural pain. Patients who reported pain in the previous 7 days were asked if stress made their pain worse (yes or no). The results showed that the belief that pain is made worse by stress was a significant predictor of severe post-procedural pain.^67^

#### Positive and negative affect

The relationship between ‘positive and negative affect’ and post-procedural pain was examined in two studies.^54,55^ Both studies used the Profile of Mood States (POMS) questionnaire prior to emergency endodontic treatment and reported no significant association between mood and post-procedural pain.

## Discussion

Although many studies in dentistry and endodontics focus on psychological constructs, particularly anxiety,^68,69^ our specific focus was on those studies that evaluated the relationship between psychological factors and endodontic pain. The current review synthesizes the results of 48 studies investigating 12 psychological constructs in relation to pre-procedural, procedural, and post-procedural pain.

### Study participants

The majority of studies were conducted in high-income countries, and over 80% of participants were recruited from university educational dental clinics/hospitals and public health centres.

### Psychological contributors to pain

Anxiety, with a focus on dental anxiety, emerged as a frequently studied psychological construct, showing a significant association with patients’ pain experiences. Previous research has also indicated a positive correlation between anxiety and pain.^47,70,71^ However, it is notable that some studies found no correlation between anxiety and pain.^31,46^

This review also underscores the significance of anticipated pain as an important psychological predictor for procedural and post-procedural pain. Perković et al.^46^ investigated the correlation between anxiety and expectations of intraoperative endodontic pain, revealing a positive link between these variables. The relationship between expected and actual pain experiences has also been documented in prior oral surgery studies.^72,73^ It is important to recognize that what patients expect before treatment can affect their experience, and this holds for different stages of treatment. Additionally, these expectations can be influenced by factors like thoughts and emotions, such as anxiety and the patient’s memory of past experiences. For instance, anticipating pain has been connected to feeling anxious about the treatment,^72,73^ and remembering the pain felt during past procedures is related to both anxiety and what was expected beforehand.^72,74^

The majority of presented constructs (11 out of 12) were related to post-procedural pain. Anxiety emerged as the most commonly studied predictor similar to pre-procedural or procedural pain with most studies reporting a positive correlation. This trend aligns with findings from a separate study involving anxiety in other dental procedures, which demonstrated a correlation between state anxiety, anticipated pain, and experienced pain.^70^ Furthermore, similar positive correlations have been observed in the context of post-procedural pain in oral surgery.^74^

Regarding the importance of depression as a psychological construct, it displays a significant relationship with both procedural and post-procedural pain in most studies. This finding is in concordance with a study in the field of oral surgery but contrasts with observations regarding pain following another oral surgery study and implant procedures maybe due to measurement scale difference.^70,75^

For the 12 constructs described, the construct of personality received limited attention with only one study^42^ addressing it. Khademi’s study,^42^ investigating the positive correlation of neurotic personality with post-procedural pain, yielded similar results to an oral surgery study.^70^ Consequently, further research in this area is warranted.

For the majority of studies reporting post-operative pain, the exact timing of assessment was either mostly short-term (immediate/within days) with two long-term studies of ∼6 months’ follow-up,^52,56^ which show psychological variables (anxiety and positive beliefs about treatment) remain relevant in predicting persistent pain. Our result is consistent with a study that showed some young people develop chronic postsurgical pain (>4 months) with distress and expectations as psychological predictors found.^76^ This supports our aim that psychological factors contribute across pain phases rather than only in acute settings.

Regarding psychological and pain measurement, inconsistencies in defining and measuring scales were evident in the reviewed studies, which may complicate any future meta-analysis; for example, dental anxiety has been measured with different scales (DAS, MDAS, DFS) without proper justification. A 2025 update on the DAS highlights limited longitudinal validity evidence, limiting the use across time.^77^ Another example would be the Mannan and Greenspan study,^45^ who utilized the Hospital Anxiety and Depression Scale (HADS) to measure depression, which increased the study’s feasibility, cost, and potential sample size. Regarding the pain scale, a recent oral-medicine review summarizes practical pros/cons of VAS, NRS, VRS, and face scales in dental settings, which can guide more consistent selection.^23^ Most studies used single-dimention pain intensity measures, VAS or NRS, which are valid for pain intensity but do not capture affective factors (like mood), which may influence observed anxiety, pain correlations.^23,78^ Another potential avenue for improvement could involve shifting the focus from measuring individual pain experiences to assessing the ‘relief of pain’ as suggested in some studies.^31,74,79^ Pain reduction and pain relief represent distinct concepts with different assessment directions. Pain relief is associated with both cortical and descending pain modulation, which supports the notion of an association between pain relief and pain behaviour.^80^ Additionally, thoughtful study design, considering multiple time points for pain measurement and consistent assessment protocols, is vital to enhance the accuracy and comprehensibility of research findings. Many of the studies reviewed assessed procedural pain after treatment, yet the exact timing was often unclear.^31^ For instance, it was unclear whether the effects of local anaesthesia had dissipated at the time of procedural pain measurement.^48^ This practice enhances the comprehensibility of research outcomes and facilitates meaningful comparisons with other investigations.

Our broad inclusion of age groups and treatment types reflects the aim of mapping the scope of available evidence rather than establishing clinical specificity. While this approach may increase heterogeneity, it provides a comprehensive overview and highlights areas where evidence is limited, and future research needed.

Despite the possibility of conducting a systematic review to elucidate the significance of psychological factors, challenges originating from study design heterogeneity and data collection methods must be addressed.

### Limitations

Limitations to this study include restricting our search to studies published in English only, and also to psychological and not social factors. We acknowledge that certain constructs, such as perceived empathy, contains social as well as psychological dimensions. We intentionally narrowed the focus to psychological factors in line with our inclusion criteria. This choice does not diminish the importance of social determinants, which need dedicated investigation in future research. The anxiety construct in this review groups together some distinct psychological variables for easier interpretation, which may oversimplify the unique contributions of each individual factor. Also differences in design or treatment protocols may affect associations between psychological factors and pain outcomes. This heterogeneity limits direct comparability; findings therefore should be interpreted with caution.

### Future direction

We found some studies working on interventions to address the psychological factors in dental pain, mostly in paediatric patients, with techniques such as distraction employing virtual reality^81^ and audio-visual video eyeglasses.^82^ While the literature offers limited insight into interventions for anxiety reduction in adult dental settings, certain strategies, including ‘tell-show-do’,^68,83,84^ hypnosis,^85^ auditory distraction through background music,^86^ and cognitive therapy,^83^ have shown some promise in the field of general dentistry. To advance the development of effective interventions, our next research step should involve a comprehensive review specifically focused on existing interventions relevant to endodontic treatment to reduce anxiety or expected pain as the most common pain contributing factors in research. Understanding the interplay between psychological factors, patient expectations, and technical complexities can contribute significantly to enhancing the patient experience and treatment outcomes in endodontic procedures. Future research should examine other constructs with limited research identified in this review, such as coping strategies, beliefs about pain, personality, and desire for control, to determine whether interventions targeting these factors could complement anxiety-focused approaches.

### Clinical Implication

Although this scoping review was not designed to establish causality, it is important to acknowledge the potential bidirectional relationship between anxiety and pain: higher pre-treatment anxiety may heighten pain, while greater pain intensity may in turn reinforce anxiety which needs more investigation in future studies. For endodontic practice, realistic intervention targets include brief chairside strategies such as routine anxiety screening, expectation setting conversations, and offering patients simple control mechanisms (e.g. stop signals) to reduce perceived helplessness.^87,88^ Evidence from adult dentistry suggest that short interventions like relaxation guidance, targeted reassurance, or cognitive techniques can be integrated into routine workflows with minimal disruption, though their evaluation in endodontics in limited.^87,89–92^ The feasibility of these interventions may depend on factors such as the limited clinical time available in general practice, as well as the level of training and expertise required for dentists to recognize patient anxiety and deliver appropriate support, so a psychology–dentistry collaboration is required for effective results.

### Conclusion

Many different psychological factors have been found that relate to pain of pre-procedural, procedural, and post-procedural endodontics. We have classified these predictors into 12 overarching constructs, among which anxiety and expected pain have emerged as the most commonly studied factors. More research is required to understand the main factors with positive correlations to pain and how to manage these psychological factors to help manage pain better in dentistry.

## Supplemental Material

Supplemental Material - Psychological contributors to pain before, during, and after endodontic procedures: A scoping review Supplemental Material for Psychological contributors to pain before, during, and after endodontic procedures: A scoping review by Atieh Sadr, Ali Gholamrezaei, Amy G. McNeilage, Cameron L. Randall, Flavia P. Kapos, Christopher C. Peck, and Claire E. Ashton-James in British Journal of Pain

## References

[1] Cunningham CJ Mullaney TP . Pain control in endodontics. Dent Clin North Am 1992; 36(2): 393–408. 1572506

[2] Sathorn C Parashos P Messer H . The prevalence of postoperative pain and flare-up in single- and multiple-visit endodontic treatment: a systematic review. Int Endod J 2008; 41(2): 91–99. 17956561 10.1111/j.1365-2591.2007.01316.x

[3] Walton R Fouad A . Endodontic interappointment flare-ups: a prospective study of incidence and related factors. J Endod 1992; 18(4): 172–177. 1402571 10.1016/S0099-2399(06)81413-5

[4] Harrison JW Baumgartner JC Svec TA . Incidence of pain associated with clinical factors during and after root canal therapy. Part 2. Postobturation pain. J Endod 1983; 9(10): 434–438. 6579169 10.1016/S0099-2399(83)80259-3

[5] Smith EA Marshall JG Selph SS , et al. Nonsteroidal anti-inflammatory drugs for managing postoperative endodontic pain in patients who present with preoperative pain: a systematic review and meta-analysis. J Endod 2017; 43(1): 7–15. 27939729 10.1016/j.joen.2016.09.010

[6] Shirvani A Shamszadeh S Eghbal MJ , et al. The efficacy of non-narcotic analgesics on post-operative endodontic pain: a systematic review and meta-analysis: the efficacy of non-steroidal anti-inflammatory drugs and/or paracetamol on post-operative endodontic pain. J Oral Rehabil 2017; 44(9): 709–721. 28449307 10.1111/joor.12519

[7] Iranmanesh F Parirokh M Haghdoost AA , et al. Effect of corticosteroids on pain relief following root canal treatment: A systematic review. Iran Endod J 2017; 12(2): 123–130. 28496516 10.22037/iej.2017.26 PMC5421265

[8] Pak JG White SN . Pain prevalence and severity before, during, and after root canal treatment: a systematic review. J Endod 2011; 37(4): 429–438. 21419285 10.1016/j.joen.2010.12.016

[9] Vlaeyen JWS Linton SJ . Fear-avoidance and its consequences in chronic musculoskeletal pain: a state of the art. Pain 2000; 85(3): 317–332. 10781906 10.1016/S0304-3959(99)00242-0

[10] Eccleston C Crombez G . Pain demands attention: a cognitive-affective model of the interruptive function of pain. Psychol Bull 1999; 125(3): 356–366. 10349356 10.1037/0033-2909.125.3.356

[11] Masaud K Galvin AD De Loughry G , et al. Preoperative psychological factors influence analgesic consumption and self-reported pain intensity following breast cancer surgery. BMC Anesthesiol 2024; 24(1): 239. 39014332 10.1186/s12871-024-02622-6 PMC11250972

[12] Giusti EM Lacerenza M Manzoni GM , et al. Psychological and psychosocial predictors of chronic postsurgical pain: a systematic review and meta-analysis. Pain 2021; 162(1): 10–30. 32694386 10.1097/j.pain.0000000000001999

[13] Terradas-Monllor M Ruiz MA Ochandorena-Acha M . Postoperative psychological predictors for chronic postsurgical pain after a knee arthroplasty: a prospective observational Study. Phys Ther 2024; 104(1): pzad141. 37831899 10.1093/ptj/pzad141

[14] Raja SN Carr DB Cohen M , et al. The revised international association for the study of pain definition of pain: concepts, challenges, and compromises. Pain 2020; 161(9): 1976–1982. 32694387 10.1097/j.pain.0000000000001939 PMC7680716

[15] Turk DC Okifuji A . Psychological factors in chronic pain: evolution and revolution. J Consult Clin Psychol 2002; 70(3): 678–690. 12090376 10.1037//0022-006x.70.3.678

[16] Che X Cash R Fitzgerald P , et al. The social regulation of pain: Autonomic and neurophysiological changes associated with perceived threat. J Pain 2018; 19(5): 496–505. 29274393 10.1016/j.jpain.2017.12.007

[17] Karran EL Grant AR Moseley GL . Low back pain and the social determinants of health: a systematic review and narrative synthesis. Pain 2020; 161(11): 2476–2493. 32910100 10.1097/j.pain.0000000000001944

[18] Thom A Sartory G Jöhren P . Comparison between one-session psychological treatment and benzodiazepine in dental phobia. J Consult Clin Psychol 2000; 68(3): 378–387. 10883554 10.1037//0022-006x.68.3.378

[19] Hruschak V Cochran G . Psychosocial predictors in the transition from acute to chronic pain: a systematic review. Psychol Health Med 2018; 23(10): 1151–1167. 29490476 10.1080/13548506.2018.1446097 PMC6708443

[20] Peters MDJ Marnie C Tricco AC , et al. Updated methodological guidance for the conduct of scoping reviews. JBI Evid Synth 2020; 18(10): 2119–2126. 33038124 10.11124/JBIES-20-00167

[21] Tricco AC Lillie E Zarin W , et al. PRISMA extension for scoping reviews (PRISMA-ScR): checklist and explanation. Ann Intern Med 2018; 169(7): 467–473. 30178033 10.7326/M18-0850

[22] Covidence Systematic Review Software. Veritas Health Innovation. https://www.covidence.org/

[23] Scribante A Pellegrini M Pulicari F , et al. Pain assessment in oral medicine through its different dimensions: a comprehensive review. Dent J 2023; 11(11): 246. 10.3390/dj11110246 PMC10670171 37999011

[24] Alroomy R Kim D Hochberg R , et al. Factors influencing pain and anxiety before endodontic treatment: a cross-sectional Study amongst American individuals. Eur Endod J 2020; 5(3): 199–204. 33353908 10.14744/eej.2020.17363 PMC7881385

[25] Brawley D . Pain relief knowledge, history, and expectations among emergency dental patients. West Virginia University, Master’s thesis, 2019.

[26] Khoo ST Ode W Lopez V , et al. Factors influencing quality of life after surgical and nonsurgical interventions of persistent endodontic disease. J Endod 2020; 46(12): 1832–1840. 32898556 10.1016/j.joen.2020.08.020

[27] Laxmi I kumar S Ramesh S . Clinical correlation of salivary alpha amylase activity versus dental anxiety levels in patients with chronic endodontic pain. Ann Med Health Sci Res 2021; 11: 62–67.

[28] Murillo-Benítez M Martín-González J Jiménez-Sánchez MC , et al. Association between dental anxiety and intraoperative pain during root canal treatment: a cross-sectional study. Int Endod J 2020; 53(4): 447–454. 31691312 10.1111/iej.13245

[29] Peretz B Moshonov J . Dental anxiety among patients undergoing endodontic treatment. J Endod 1998; 24(6): 435–437. 9693590 10.1016/S0099-2399(98)80028-9

[30] Sorrell JT . Effects of fear of dental pain and information type on fear and pain responding during endodontic treatment. West Virginia University, 2003.

[31] Wu LT Lin CS Yang SF . Association between pain, anxiety, and pain relief in patients receiving emergent endodontic treatment. Clin Oral Investig 2022; 26(1): 275–285. 10.1007/s00784-021-03997-3 34080062

[32] de Oliveira JP de Alencar AHG Estrela CB , et al. Comparative effectiveness of preemptive administration of ibuprofen and ibuprofen-arginine on the anesthetic success of inferior alveolar nerve block in teeth with symptomatic irreversible pulpitis - a double-blind randomized clinical trial. Clin Oral Investig 2024; 28(7): 366. 10.1007/s00784-024-05765-5 38850383

[33] Gastmann AH Xavier SR Pilownic KJ , et al. Pain, anxiety, and catastrophizing among pregnant women with dental pain, undergoing root canal treatment. Braz Oral Res 2024; 38: e054. 38922214 10.1590/1807-3107bor-2024.vol38.0054 PMC11376644

[34] Arntz A van Eck M Heijmans M . Predictions of dental pain: the fear of any expected evil, is worse than the evil itself. Behav Res Ther 1990; 28(1): 29–41. 2302147 10.1016/0005-7967(90)90052-k

[35] Attar RH Baghdadi ZD . Comparative efficacy of active and passive distraction during restorative treatment in children using an iPad versus audiovisual eyeglasses: a randomised controlled trial. Eur Arch Paediatr Dent 2015; 16(1): 1–8. 25416522 10.1007/s40368-014-0136-x

[36] Chandraweera L Goh K Lai-Tong J , et al. A survey of patients’ perceptions about, and their experiences of, root canal treatment. Aust Endod J 2019; 45(2): 225–232. 30341798 10.1111/aej.12312

[37] Cho SY Kim E Park SH , et al. Effect of topical Anesthesia on pain from needle insertion and injection and its relationship with anxiety in patients awaiting apical surgery: a randomized double-blind clinical trial. J Endod 2017; 43(3): 364–369. 28110919 10.1016/j.joen.2016.10.036

[38] Dou L Vanschaayk MM Zhang Y , et al. The prevalence of dental anxiety and its association with pain and other variables among adult patients with irreversible pulpitis. BMC Oral Health 2018; 18(1): 101. 29879974 10.1186/s12903-018-0563-x PMC5992818

[39] Eli I Bar-Tal Y Fuss Z , et al. Effect of intended treatment on anxiety and on reaction to electric pulp stimulation in dental patients. J Endod 1997; 23(11): 694–697. 9587311 10.1016/S0099-2399(97)80404-9

[40] Georgelin-Gurgel M Diemer F Nicolas E , et al. Surgical and nonsurgical endodontic treatment-induced stress. J Endod 2009; 35(1): 19–22. 19084118 10.1016/j.joen.2008.09.019

[41] Gochenour LL . Cortisol responsivity: Association with fear and pain related to root canal therapy. Master’s thesis, West Virginia University Libraries, Morgantown, WV, 2003.

[42] Khademi A Roohafza H Iranmanesh P , et al. Association between psychological factors and pain perception in patients with symptomatic irreversible pulpitis during endodontic treatment. G Ital Endod 2021; 35(1): 7.

[43] Klages U Ulusoy O Kianifard S , et al. Dental trait anxiety and pain sensitivity as predictors of expected and experienced pain in stressful dental procedures. Eur J Oral Sci 2004; 112(6): 477–483. 15560829 10.1111/j.1600-0722.2004.00167.x

[44] LeClaire AJ Skidmore AE Griffin JA Jr , et al. Endodontic fear survey. J Endod 1988; 14(11): 560–564. 3249195 10.1016/S0099-2399(88)80091-8

[45] Mannan S . Observational study evaluating pain in endodontic patients diagnosed with depression – a pilot study. MD, USA: University of Maryland School of Dentistry, 2019.

[46] Perković I Romić MK Perić M , et al. The level of anxiety and pain perception of endodontic patients. Acta Stomatol Croat 2014; 48(4): 258–267. 27688374 10.15644/asc47/4/3 PMC4872819

[47] Watkins CA Logan HL Kirchner HL . Anticipated and experienced pain associated with endodontic therapy. J Am Dent Assoc 2002; 133(1): 45–54. 11811742 10.14219/jada.archive.2002.0020

[48] Weitz D . Preoperative factors associated with anesthesia failure for patients undergoing non-surgical root canal therapy; a national dental practice-based research network DPBRN study. University of Minnesota. Digital Conservancy, 2020. 10.1016/j.joen.2021.09.005 PMC8629850 34560117

[49] Nogueira APA Ferreira MC Maia CCR , et al. Efficacy of articaine anesthesia with needle-free/comfort-in method and conventional needle injection in dental patients with irreversible pulpitis: a randomized clinical trial. Clin Oral Investig 2024; 28(3): 205. 10.1007/s00784-024-05582-w 38459266

[50] Silva IA Agnol Júnior CAD Weissheimer T , et al. Pharmacological management of anxiety on pain occurrence during root canal treatment: a systematic review. Eur Endod J 2023; 8(2): 105–113. 37010201 10.14744/eej.2022.83097 PMC10098429

[51] Baron RS Logan H Hoppe S . Emotional and sensory focus as mediators of dental pain among patients differing in desired and felt dental control. Health Psychol 1993; 12(5): 381–389. 8223362 10.1037//0278-6133.12.5.381

[52] Daline IH Nixdorf DR Law AS , et al. 3-year outcome of patients with persistent pain after Root canal treatment: the national dental practice-based research network. J Endod 2020; 46(5): 619–626. e2. 32171563 10.1016/j.joen.2020.01.018 PMC7217349

[53] Edwards RR Fillingim RB Maixner W , et al. Catastrophizing predicts changes in thermal pain responses after resolution of acute dental pain. J Pain 2004; 5(3): 164–170. 15106129 10.1016/j.jpain.2004.02.226

[54] Edwards RR Fillingim RB Yamauchi S , et al. Effects of gender and acute dental pain on thermal pain responses. Clin J Pain 1999; 15(3): 233–237. 10524477 10.1097/00002508-199909000-00011

[55] Mohorn S Maizner W Fillingim R , et al. Effect of psychological-factors on preoperative and postoperative endodontic pain. Journal of Dental Research 1995; 74: 22314. AMER Assoc Dental Research 1619 DUKE ST.

[56] Nixdorf DR Law AS Lindquist K , et al. Frequency, impact, and predictors of persistent pain after root canal treatment: a national dental PBRN study. Pain 2016; 157(1): 159–165. 26335907 10.1097/j.pain.0000000000000343 PMC4684798

[57] Philpott R Gulabivala K Leeson R , et al. Prevalence, predictive factors and clinical course of persistent pain associated with teeth displaying periapical healing following nonsurgical root canal treatment: a prospective study. Int Endod J 2019; 52(4): 407–415. 30332512 10.1111/iej.13029

[58] Pillai RS Pigg M List T , et al. Assessment of somatosensory and psychosocial function of patients with trigeminal nerve damage. Clin J Pain 2020; 36(5): 321–335. 31977376 10.1097/AJP.0000000000000806

[59] Rahman N Fullen B Canavan D , et al. T252 dental POST procedural pain: impact of demographic factors. European Journal of Pain Supplements 2011; 5(5): 51.

[60] Yang SE Park YG Han K , et al. Association between dental pain and depression in Korean adults using the Korean National Health and Nutrition Examination Survey. J Oral Rehabil 2016; 43(1): 51–58. 26337763 10.1111/joor.12343

[61] Applebaum EA Maixner W . Genetic basis for individual variation in pain perception among endodontic patients . Masters Thesis. 2009.

[62] Ahmed S Sharma P Mahaprasad A , et al. Assessing the influence of patient anxiety on the efficacy of endodontic procedures. J Pharm BioAllied Sci 2024; 16(Suppl 3): S2685–S2687. 39346408 10.4103/jpbs.jpbs_371_24 PMC11426610

[63] Mijailovic M Kolak V . The influence of psychological factors on the frequency and perception of post-endodontic pain. Vojnosanit Pregl 2024; 81(4): 212–219.

[64] Vitali FC Mafra G Santos PS , et al. Patient-related predictors of post-operative pain following root canal treatment: a structural model analysis. Int Endod J 2024; 57(12): 1758–1768. 39150401 10.1111/iej.14137

[65] Maggirias J Locker D . Psychological factors and perceptions of pain associated with dental treatment. Community Dent Oral Epidemiol 2002; 30(2): 151–159. 12000356 10.1034/j.1600-0528.2002.300209.x

[66] Miura A Tu TTH Shinohara Y , et al. Psychiatric comorbidities in patients with Atypical Odontalgia. J Psychosom Res 2018; 104: 35–40. 29275783 10.1016/j.jpsychores.2017.11.001

[67] Law AS Nixdorf DR Aguirre AM , et al. Predicting severe pain after root canal therapy in the National dental PBRN. J Dent Res 2014; 94(3_suppl): 37S–43S. 25355775 10.1177/0022034514555144 PMC4336154

[68] Wide Boman U Carlsson V Westin M , et al. Psychological treatment of dental anxiety among adults: a systematic review. Eur J Oral Sci 2013; 121(3 Pt 2): 225–234. 23659254 10.1111/eos.12032

[69] Khan S Hamedy R Lei Y , et al. Anxiety related to nonsurgical root canal treatment: A systematic review. J Endod 2016; 42(12): 1726–1736. 27776881 10.1016/j.joen.2016.08.007

[70] Feinmann C Ong M Harvey W , et al. Psychological factors influencing post-operative pain and analgesic consumption. Br J Oral Maxillofac Surg 1987; 25(4): 285–292. 3476153 10.1016/0266-4356(87)90067-2

[71] Farias Z Campello CP da Silveira MMF , et al. The influence of anxiety on pain perception and its repercussion on endodontic treatment: a systematic review. Clin Oral Investig 2023; 27(10): 5709–5718. 10.1007/s00784-023-05181-1 37526740

[72] Gómez-de Diego R Cutando-Soriano A Montero-Martín J , et al. State anxiety and depression as factors modulating and influencing postoperative pain in dental implant surgery. A prospective clinical survey. Med Oral Patol Oral Cir Bucal 2014; 19(6): e592–e597. 24880447 10.4317/medoral.19685 PMC4259376

[73] Treister R Eaton TA Trudeau JJ , et al. Development and preliminary validation of the focused analgesia selection test to identify accurate pain reporters. J Pain Res 2017; 10: 319–326. 28243138 10.2147/JPR.S121455 PMC5315353

[74] Navratilova E Porreca F . Reward and motivation in pain and pain relief. Nat Neurosci 2014; 17(10): 1304–1312. 25254980 10.1038/nn.3811 PMC4301417

[75] Dadgostar A Bigder M Punjani N , et al. Does preoperative depression predict post-operative surgical pain: a systematic review. Int J Surg 2017; 41: 162–173. 28359955 10.1016/j.ijsu.2017.03.061

[76] Rabbitts JA Palermo TM Zhou C , et al. Psychosocial predictors of acute and chronic pain in adolescents undergoing major musculoskeletal surgery. J Pain 2020; 21(11-12): 1236–1246. 32553622 10.1016/j.jpain.2020.02.004 PMC7721978

[77] Bantel D Chmielewski WX Brähler E , et al. The dental anxiety scale (DAS) – psychometric properties and longitudinal findings among middle-aged adults. BMC Psychol 2025; 13(1): 953. 40842032 10.1186/s40359-025-03304-9 PMC12372296

[78] Hjermstad MJ Fayers PM Haugen DF , et al. Studies comparing numerical rating scales, verbal rating scales, and visual analogue scales for assessment of pain intensity in adults: a systematic literature review. J Pain Symptom Manage 2011; 41(6): 1073–1093. 21621130 10.1016/j.jpainsymman.2010.08.016

[79] Leknes S Berna C Lee MC , et al. The importance of context: when relative relief renders pain pleasant. Pain 2013; 154(3): 402–410. 23352758 10.1016/j.pain.2012.11.018 PMC3590449

[80] Leknes S Brooks JC Wiech K , et al. Pain relief as an opponent process: a psychophysical investigation. Eur J Neurosci 2008; 28(4): 794–801. 18671736 10.1111/j.1460-9568.2008.06380.x

[81] Yan X Yan Y Cao M , et al. Effectiveness of virtual reality distraction interventions to reduce dental anxiety in paediatric patients: a systematic review and meta-analysis. J Dent 2023; 132: 104455. 36842625 10.1016/j.jdent.2023.104455

[82] Ram D Shapira J Holan G , et al. Audiovisual video eyeglass distraction during dental treatment in children. Quintessence Int 2010; 41(8): 673–679. 20657857

[83] Berggren U Hakeberg M Carlsson SG . Relaxation vs. cognitively oriented therapies for dental fear. J Dent Res 2000; 79(9): 1645–1651. 11023258 10.1177/00220345000790090201

[84] Allen KD Stanley RT McPherson K . Evaluation of behavior management technology dissemination in pediatric dentistry. Pediatr Dent 1990; 12(2): 79–82. 2151957

[85] Glaesmer H Geupel H Haak R . A controlled trial on the effect of hypnosis on dental anxiety in tooth removal patients. Patient Educ Couns 2015; 98(9): 1112–1115. 26054452 10.1016/j.pec.2015.05.007

[86] Moola S Pearson A Hagger C . Effectiveness of music interventions on dental anxiety in paediatric and adult patients: a systematic review. JBI Libr Syst Rev 2011; 9(18): 588–630. 27819961 10.11124/01938924-201109180-00001

[87] Hoffmann B Erwood K Ncomanzi S , et al. Management strategies for adult patients with dental anxiety in the dental clinic: a systematic review. Aust Dent J 2022; 67(Suppl 1): S3–s13. 35735746 10.1111/adj.12926 PMC9796536

[88] Appukuttan DP . Strategies to manage patients with dental anxiety and dental phobia: literature review. Clin Cosmet Investig Dent 2016; 8: 35–50. 10.2147/CCIDE.S63626 PMC4790493 27022303

[89] Alsibai E Bshara N Alzoubi H , et al. Assessing an active distracting technique during primary mandibular molar pulpotomy (randomized controlled trial). Clin Exp Dent Res 2023; 9(2): 283–289. 36478192 10.1002/cre2.702 PMC10098273

[90] Craveiro MA Caldeira CL . Influence of An audiovisual resource on the preoperative anxiety of adult endodontic patients: a randomized controlled clinical trial. J Endod 2020; 46(7): 909–914. 32389383 10.1016/j.joen.2020.03.024

[91] Elmowitz JS Shupak RP . Pharmacological and non-pharmacological methods of postoperative pain control following oral and maxillofacial surgery: a scoping review. J Oral Maxillofac Surg 2021; 79(10): 2000–2009. 34097866 10.1016/j.joms.2021.04.022

[92] Doan LV Blitz J . Preoperative assessment and management of patients with pain and anxiety disorders. Curr Anesthesiol Rep 2020; 10(1): 28–34. 32435161 10.1007/s40140-020-00367-9 PMC7222996

