# Supplementary material for: Psychological contributors to pain before, during, and after endodontic procedures: A scoping review
Source: Br J Pain. 2025 Dec 26:20494637251408718. Online ahead of print. doi: 10.1177/20494637251408718 (PMC12743013; doi:10.1177/20494637251408718)
Supplement: Supplemental Material - Psychological contributors to pain before, during, and after endodontic procedures: A scoping review [file sj-pdf-1-bjp-10.1177_20494637251408718.pdf]

Psychological Contributors to Pain Before, During, and After Endodontic Procedures: A Scoping Review

Appendix 1: Databases search codes

PubMed

(pain OR pains OR painful* OR headache* OR cephalalg* OR cephalg* OR cephalodynia* OR glossodynia* OR glossalg* OR glossopyrosis OR toothache* OR tooth-ache* OR odontalg* OR neuralg* OR neurodynia OR earache* OR ear-ache* OR otalg* OR hypersens* OR hyperalg* OR allodyn* OR "tooth sensitivity" OR "teeth sensitivity" OR "dental sensitivity" OR "dentin sensitivity" OR ache OR aches OR aching OR soreness OR sore OR flare-up* OR flareup* OR Pain[Mesh] OR Pain Measurement[Mesh] OR Hyperalgesia[Mesh] OR Dentin Sensitivity[Mesh] OR Neuralgia[Mesh] OR Symptom Flare Up[Mesh]) AND (psych* OR emotion* OR emotive OR cognition* OR cognitive* OR behavior* OR behaviour* OR mental* OR motivation* OR expectanc* OR expectation* OR anticipat* OR memory OR memories OR attitude* OR belief* OR appraisal* OR uncertaint* OR predictability OR controllability OR helplessness OR self-efficacy OR perceived-control OR felt-control OR "locus of control" OR "loss of control" OR "positive experience*" OR "negative experience*" OR threat* OR feeling* OR mood* OR fear* OR anxiet* OR anxious* OR hypervigilant* OR depression OR depressive* OR guilt* OR shame* OR embarrassment* OR distress* OR stress* OR worry OR worrisome OR disgust* OR "negative affect*" OR "positive affect*" OR "affective state*" OR phobi* OR odontophobia OR panic OR concern OR concerns OR avoidance OR reward* OR reinforc* OR vicarious* OR coping* OR learning* OR adaptation* OR optimism OR pessimism OR somatization OR somatisation OR somatoform OR neuroticism OR catastrophiz* OR catastrophis* OR nervous* OR personalit* OR mindful* OR Psychiatry and Psychology Category[Mesh] OR Health Belief Model[Mesh]) AND (Endodontic* OR (("root canal*") AND (therapy OR procedure OR treatment OR retreatment OR management OR surgery OR filling OR debridement OR devitalization OR obturation OR preparation OR resection OR hemisection)) OR pulpectomy OR pulpotomy OR (pulp AND (revascularization OR regeneration OR capping)) OR "apical surgery" OR apicoectomy OR ((peri-radicular OR periradicular) AND surgery) OR Apexification OR Apexogenesis OR Endodontics[Mesh])

OVID (EMBASE)

(pain OR pains OR painful* OR headache* OR cephalalg* OR cephalg* OR cephalodynia* OR glossodynia* OR glossalg* OR glossopyrosis OR toothache* OR tooth-ache* OR odontalg* OR neuralg* OR neurodynia OR earache* OR ear-ache* OR otalg* OR hypersens* OR hyperalg* OR allodyn* OR tooth sensitivity OR teeth sensitivity OR dental sensitivity OR dentin sensitivity OR ache OR aches OR aching OR soreness OR sore OR flare-up* OR flareup* OR exp Pain/ OR exp Pain Measurement/ OR exp Hyperesthesia/ OR exp Dentin Sensitivity/ OR exp Neuralgia/) AND (emotion* OR emotive OR cognition* OR cognitive* OR behavior* OR behaviour* OR mental* OR motivation* OR expectanc* OR expectation* OR anticipat* OR memory OR memories OR attitude* OR belief* OR appraisal* OR uncertaint* OR predictability OR controllability OR helplessness OR self-efficacy OR perceived control OR felt control OR locus of control OR loss of control OR positive experience* OR negative experience* OR threat* OR feeling* OR mood* OR fear* OR anxiet* OR anxious* OR hypervigilant* OR depression OR depressive* OR guilt* OR shame* OR embarrassment* OR distress* OR stress* OR worry OR worrisome OR disgust* OR negative affect* OR positive affect* OR affective state* OR phobi* OR odontophobia OR panic OR concern OR concerns OR avoidance OR reward* OR reinforc* OR vicarious* OR coping* OR learning* OR adaptation* OR optimism OR pessimism OR somatization OR somatisation OR somatoform OR neuroticism OR catastrophiz* OR catastrophis* OR nervous* OR personalit* OR mindful* OR exp psychiatry/ OR exp behavioral science/ OR exp behavior/) AND (Endodontic* OR (root canal* AND (therapy OR procedure OR treatment OR retreatment OR management OR surgery OR filling OR debridement OR devitalization OR obturation OR preparation OR resection OR hemisection)) OR pulpectomy OR pulpotomy OR (pulp AND (revascularization OR regeneration OR capping)) OR apical surgery OR apicoectomy OR ((peri-radicular OR periradicular) AND surgery) OR Apexification OR Apexogenesis OR exp Endodontics/)

OVID (PsycINFO)

(pain OR pains OR painful* OR headache* OR cephalalg* OR cephalg* OR cephalodynia* OR glossodynia* OR glossalg* OR glossopyrosis OR toothache* OR tooth-ache* OR odontalg* OR neuralg* OR neurodynia OR earache* OR ear-ache* OR otalg* OR hypersens* OR hyperalg* OR allodyn* OR tooth sensitivity OR teeth sensitivity OR dental sensitivity OR dentin sensitivity OR ache OR aches OR aching OR soreness OR sore OR flare-up* OR flareup* OR exp Pain/ OR exp Pain Measurement/ OR exp Hyperalgesia/ OR exp Hyperesthesia/ OR exp Hypesthesia/ OR exp Paresthesia/) AND (psych* OR emotion* OR emotive OR cognition* OR cognitive* OR behavior* OR behaviour* OR mental* OR motivation* OR expectanc* OR expectation* OR anticipat* OR memory OR memories OR attitude* OR belief* OR appraisal* OR uncertaint* OR predictability OR controllability OR helplessness OR self-efficacy OR perceived control OR felt control OR locus of control OR loss of control OR positive experience* OR negative experience* OR threat* OR feeling* OR mood* OR fear* OR anxiet* OR anxious* OR hypervigilant* OR depression OR depressive* OR guilt* OR shame* OR embarrassment* OR distress* OR stress* OR worry OR worrisome OR disgust* OR negative affect* OR positive affect* OR affective state* OR phobi* OR odontophobia OR panic OR concern OR concerns OR avoidance OR reward* OR reinforc* OR vicarious* OR coping* OR learning* OR adaptation* OR optimism OR pessimism OR somatization OR somatisation OR somatoform OR neuroticism OR catastrophiz* OR catastrophis* OR nervous* OR personalit* OR mindful* OR exp Behavioral Sciences/) AND (Endodontic* OR (root canal* AND (therapy OR procedure OR treatment OR retreatment OR management OR surgery OR filling OR debridement OR devitalization OR obturation OR preparation OR resection OR hemisection)) OR pulpectomy OR pulpotomy OR (pulp AND (revascularization OR regeneration OR capping)) OR apical surgery OR apicoectomy OR ((peri-radicular OR periradicular) AND surgery) OR Apexification OR Apexogenesis)

OVID (Cochrane Database of Systematic Reviews, Cochrane Central Register of Controlled Trials)

((pain OR pains OR painful* OR headache* OR cephalalg* OR cephalg* OR cephalodynia* OR glossodynia* OR glossalg* OR glossopyrosis OR toothache* OR tooth-ache* OR odontalg* OR neuralg* OR neurodynia OR earache* OR ear-ache* OR otalg* OR hypersens* OR hyperalg* OR allodyn* OR tooth sensitivity OR teeth sensitivity OR dental sensitivity OR dentin sensitivity OR ache OR aches OR aching OR soreness OR sore OR flare-up* OR flareup*) OR (exp Pain/ OR exp Somatosensory Disorders/ OR exp Neuralgia/)) AND ((psych* OR emotion* OR emotive OR cognition* OR cognitive* OR behavior* OR behaviour* OR mental* OR motivation* OR expectanc* OR expectation* OR anticipat* OR memory OR memories OR attitude* OR belief* OR appraisal* OR uncertaint* OR predictability OR controllability OR helplessness OR self-efficacy OR perceived control OR felt control OR locus of control OR loss of control OR positive experience* OR negative experience* OR threat* OR feeling* OR mood* OR fear* OR anxiet* OR anxious* OR hypervigilant* OR depression OR depressive* OR guilt* OR shame* OR embarrassment* OR distress* OR stress* OR worry OR worrisome OR disgust* OR negative affect* OR positive affect* OR affective state* OR phobi* OR odontophobia OR panic OR concern OR concerns OR avoidance OR reward* OR reinforc* OR vicarious* OR coping* OR learning* OR adaptation* OR optimism OR pessimism OR somatization OR somatisation OR somatoform OR neuroticism OR catastrophiz* OR catastrophis* OR nervous* OR personalit* OR mindful*) OR (exp Behavioral Disciplines and Activities/)) AND ((Endodontic* OR (root canal* AND (therapy OR procedure OR treatment OR retreatment OR management OR surgery OR filling OR debridement OR devitalization OR obturation OR preparation OR resection OR hemisection)) OR pulpectomy OR pulpotomy OR (pulp AND (revascularization OR regeneration OR capping)) OR apical surgery OR apicoectomy OR ((peri-radicular OR periradicular) AND surgery) OR Apexification OR Apexogenesis) OR (exp Endodontics/))

CINAHL

(pain OR pains OR "painful* OR headache*" OR cephalalg* OR cephalg* OR cephalodynia* OR glossodynia* OR glossalg* OR glossopyrosis OR toothache* OR tooth-ache* OR odontalg* OR neuralg* OR neurodynia OR earache* OR ear-ache* OR otalg* OR hypersens* OR hyperalg* OR allodyn* OR "tooth sensitivity" OR "teeth sensitivity" OR "dental sensitivity" OR "dentin sensitivity" OR ache OR aches OR aching OR soreness OR sore OR flare-up* OR flareup* OR (MH Pain+) OR (MH "Pain Measurement+") OR (MH Hyperalgesia+) OR (MH Allodynia+) OR (MH Hyperesthesia+) OR (MH Hypesthesia+) OR (MH Paresthesia+) OR (MH "Dentin Sensitivity+")) AND (psych* OR emotion* OR emotive OR cognition* OR cognitive* OR behavior* OR behaviour* OR mental* OR motivation* OR expectanc* OR expectation* OR anticipat* OR memory OR memories OR attitude* OR belief* OR appraisal* OR uncertaint* OR predictability OR controllability OR helplessness OR "self efficacy" OR self-efficacy OR "perceived control" OR "felt control" OR "locus of control" OR "loss of control" OR "positive experience*" OR "negative experience*" OR threat* OR feeling* OR mood* OR fear* OR anxiet* OR anxious* OR hypervigilant* OR depression OR depressive* OR guilt* OR shame* OR embarrassment* OR distress* OR stress* OR worry OR worrisome OR disgust* OR "negative affect*" OR "positive affect*" OR "affective state*" OR phobi* OR odontophobia OR panic OR concern OR concerns OR avoidance OR reward* OR reinforc* OR vicarious* OR coping* OR learning* OR adaptation* OR optimism OR pessimism OR somatization OR somatisation OR somatoform OR neuroticism OR catastrophiz* OR catastrophis* OR nervous* OR personalit* OR mindful* OR (MH Behavioral Sciences+) OR (MH "Health Belief Model+")) AND (Endodontic* OR (("root canal*") AND (therapy OR procedure OR treatment OR retreatment OR management OR surgery OR filling OR debridement OR devitalization OR obturation OR preparation OR resection OR hemisection)) OR pulpectomy OR pulpotomy OR (pulp AND (revascularization OR regeneration OR capping)) OR "apical surgery" OR apicoectomy OR ((peri-radicular OR periradicular) AND surgery) OR Apexification OR Apexogenesis OR (MH Endodontics+))

Web of Science - All Databases + Conference Proceedings Citation Index

TS=((pain OR pains OR painful* OR headache* OR cephalalg* OR cephalg* OR cephalodynia* OR glossodynia* OR glossalg* OR glossopyrosis OR toothache* OR tooth-ache* OR odontalg* OR neuralg* OR neurodynia OR earache* OR ear-ache* OR otalg* OR hypersens* OR hyperalg* OR allodyn* OR "tooth sensitivity" OR "teeth sensitivity" OR "dental sensitivity" OR "dentin sensitivity" OR ache OR aches OR aching OR soreness OR sore OR flare-up* OR flareup*) AND (psych* OR emotion* OR emotive OR cognition* OR cognitive* OR behavior* OR behaviour* OR mental* OR motivation* OR expectanc* OR expectation* OR anticipat* OR memory OR memories OR attitude* OR belief* OR appraisal* OR uncertaint* OR predictability OR controllability OR helplessness OR self-efficacy OR "perceived control" OR "felt control" OR "locus of control" OR "loss of control" OR "positive experience*" OR "negative experience*" OR threat* OR feeling* OR mood* OR fear* OR anxiet* OR anxious* OR hypervigilant* OR depression OR depressive* OR guilt* OR shame* OR embarrassment* OR distress* OR stress* OR worry OR worrisome OR disgust* OR "negative affect*" OR "positive affect*" OR "affective state*" OR phobi* OR odontophobia OR panic OR concern OR concerns OR avoidance OR reward* OR reinforc* OR vicarious* OR coping* OR learning* OR adaptation* OR optimism OR pessimism OR somatization OR somatisation OR somatoform OR neuroticism OR catastrophiz* OR catastrophis* OR nervous* OR personalit* OR mindful*) AND (Endodontic* OR (("root canal*") AND (therapy OR procedure OR treatment OR retreatment OR management OR surgery OR filling OR debridement OR devitalization OR obturation OR preparation OR resection OR hemisection)) OR pulpectomy OR pulpotomy OR (pulp AND (revascularization OR regeneration OR capping)) OR "apical surgery" OR apicoectomy OR ((peri-radicular OR periradicular) AND surgery) OR Apexification OR Apexogenesis))

Scopus – only in TITLE-ABS-KEY + CONFERENCE

(pain OR pains OR painful* OR headache* OR cephalalg* OR cephalg* OR cephalodynia* OR glossodynia* OR glossalg* OR glossopyrosis OR toothache* OR tooth-ache* OR odontalg* OR neuralg* OR neurodynia OR earache* OR ear-ache* OR otalg* OR hypersens* OR hyperalg* OR allodyn* OR "tooth sensitivity" OR "teeth sensitivity" OR "dental sensitivity" OR "dentin sensitivity" OR ache OR aches OR aching OR soreness OR sore OR flare-up* OR flareup*) AND (psych* OR emotion* OR emotive OR cognition* OR cognitive* OR behavior* OR behaviour* OR mental* OR motivation* OR expectanc* OR expectation* OR anticipat* OR memory OR memories OR attitude* OR belief* OR appraisal* OR uncertaint* OR predictability OR controllability OR helplessness OR "self efficacy" OR self-efficacy OR "perceived control" OR "felt control" OR "locus of control" OR "loss of control" OR "positive experience*" OR "negative experience*" OR threat* OR feeling* OR mood* OR fear* OR anxiet* OR anxious* OR hypervigilant* OR depression OR depressive* OR guilt* OR shame* OR embarrassment* OR distress* OR stress* OR worry OR worrisome OR disgust* OR "negative affect*" OR "positive affect*" OR "affective state*" OR phobi* OR odontophobia OR panic OR concern OR concerns OR avoidance OR reward* OR reinforc* OR vicarious* OR coping* OR learning* OR adaptation* OR optimism OR pessimism OR somatization OR somatisation OR somatoform OR neuroticism OR catastrophiz* OR catastrophis* OR nervous* OR personalit* OR mindful*) AND (Endodontic* OR (("root canal*") AND (therapy OR procedure OR treatment OR retreatment OR management OR surgery OR filling OR debridement OR devitalization OR obturation OR preparation OR resection OR hemisection)) OR pulpectomy OR pulpotomy OR (pulp AND (revascularization OR regeneration OR capping)) OR "apical surgery" OR apicoectomy OR ((peri-radicular OR periradicular) AND surgery) OR Apexification OR Apexogenesis)

ProQuest,

noft((pain OR pains OR painful* OR headache* OR cephalalg* OR cephalg* OR cephalodynia* OR glossodynia* OR glossalg* OR glossopyrosis OR toothache* OR tooth-ache* OR odontalg* OR neuralg* OR neurodynia OR earache* OR ear-ache* OR otalg* OR hypersens* OR hyperalg* OR allodyn* OR "tooth sensitivity" OR "teeth sensitivity" OR "dental sensitivity" OR "dentin sensitivity" OR ache OR aches OR aching OR soreness OR sore OR flare-up* OR flareup*) AND (psych* OR emotion* OR emotive OR cognition* OR cognitive* OR behavior* OR behaviour* OR mental* OR motivation* OR expectanc* OR expectation* OR anticipat* OR memory OR memories OR attitude* OR belief* OR appraisal* OR uncertaint* OR predictability OR controllability OR helplessness OR self-efficacy OR perceived-control OR felt-control OR "locus of control" OR "loss of control" OR "positive experience*" OR "negative experience*" OR threat* OR feeling* OR mood* OR fear* OR anxiet* OR anxious* OR hypervigilant* OR depression OR depressive* OR guilt* OR shame* OR embarrassment* OR distress* OR stress* OR worry OR worrisome OR disgust* OR "negative affect*" OR "positive affect*" OR "affective state*" OR phobi* OR odontophobia OR panic OR concern OR concerns OR avoidance OR reward* OR reinforc* OR vicarious* OR coping* OR learning* OR adaptation* OR optimism OR pessimism OR somatization OR somatisation OR somatoform OR neuroticism OR catastrophiz* OR catastrophis* OR nervous* OR personalit* OR mindful*) AND (Endodontic* OR (("root canal*") AND (therapy OR procedure OR treatment OR retreatment OR management OR surgery OR filling OR debridement OR devitalization OR obturation OR preparation OR resection OR hemisection)) OR pulpectomy OR pulpotomy OR (pulp AND (revascularization OR regeneration OR capping)) OR "apical surgery" OR apicoectomy OR ((peri-radicular OR periradicular) AND surgery) OR Apexification OR Apexogenesis))

Appendix 2:
Scales used for measurement of psychological variables:

Anxiety measurement for Pre-procedural pain:

1. State Anxiety: Measured using State-Trait Anxiety Inventory (STAI).
2. Dental Anxiety: Measured in five studies using different scales, including:
   - NRS (Numeric Rating Scale)
   - DAS (Dental Anxiety Scale)

Anxiety measurement scales related to procedural pain:

1. Anxiety:
   - STAI (State-Trait Anxiety Inventory)
   - NRS (Numeric Rating Scale)
   - CARS (Clinical anxiety rating scale)
2. Dental Fear:
   - DFS (Dental Fear Survey)
3. Dental Anxiety:
   - DAS4 (Dental Anxiety Scale 4)
   - MDAS (Modified Dental Anxiety Scale)
   - SDAI (Spielberger Dental Anxiety Inventory)
   - VAS (Visual Analog Scale)

- Questionnaires (Specific questionnaires designed to measure dental anxiety)
4. Discomfort and Stress:
   - VAS (Visual Analog Scale)

Anxiety scales related to post-procedural pain:
1. Anxiety:
   - State and Trait Anxiety:
     - Numerical Rating Scale (NRS)
     - HADS (Hospital Anxiety and Depression Scale)
2. Dental Anxiety:
   - 11-point NRS (Numerical Rating Scale)
   - Graded Chronic Pain Scale (GCPS)
   - Questionnaire (Specific questionnaires designed to measure dental anxiety)
3. Pain Catastrophizing:
   - Pain Catastrophizing Scale (PCS)
   - Catastrophizing subscale of the Pain Coping Scale (PCS)
4. Oral Health-Related Anxiety:
   - Oral Health Impact Profile Questionnaire (OHIP-14)
   - PHQ-4, PHQ-15, and OHIP-49 (Specific questionnaires measuring discomfort, somatic symptoms, and psychological disability)
5. Stress:
   - A question in a national survey (specific details about the question are not provided)

Expected pain scales for procedural and post-procedural:
1. Visual Analogue Scales (VAS): VAS was one of the tools used to measure expected pain.
2. Numerical Rating Scales (NRS): NRS was another tool used to assess expected pain.
3. Pain Expectation Scale (PES): The Pain Expectation Scale was used as a specific scale to measure pain expectation.

Depression scales for procedural and post-procedural:
1. Hospital Anxiety and Depression Scale (HADS): This scale was used in multiple studies to measure depression.
2. Beck Depression Inventory-II: This scale was used by (Pillai et al.) to assess depression.
3. Questionnaire: A questionnaire was used by Yang to measure depression.

‘Positive treatment expectation’ scales for procedural and post-procedural:
1. Questionnaire: A questionnaire was used by (Daline et al.) to assess patients' optimism about the treatment outcome.
2. Numerical Rating Scale (NRS): NRS was used by (Wu et al.) to measure expected pain relief in patients undergoing emergency endodontic treatment.

Personality in just procedural pain:
"short form of the NEO Five-Factor Inventory scale."

‘Desire for Control over Dental Treatment’ for just post-procedural pain:
- Iowa Dental Control Index (ICDI): Used to assess the desire for control over dental treatment and felt control.

Perception of Dentist for just postprocedural pain:
- Baseline question to assess whether patients had ever had a painful, frightening, or embarrassing experience with a dentist: Used by (Maggiria and Locker).

- Assessment of the perceived empathy of the dentist during root canal treatment: Used by (Perkovic et al).

‘Somatic Focus or Awareness’ for just postprocedural pain:
- Patient Health Questionnaire 15 (PHQ-15): Used by (Pillai et al.) to measure somatic symptom severity.
- Pennebaker Inventory of Limbic Languidness (PILL): Used by Applebaum and Maixner to measure somatization.
- National survey to measure melancholy, consultation with a psychiatrist, and suicidal thoughts: Used by (Yang et al).

‘Pain Coping Strategies’ for just postprocedural pain:
- Coping Strategies Questionnaire (CSQ): Used in two studies to measure pain coping strategies before emergency endodontic treatment.

‘Beliefs about Pain’ for just post-procedural pain:
- Questionnaire assessing the relationship between "stress that makes the pain worse" and severe post-procedural pain: Used by Law.

‘Positive and Negative Effects’ for just postprocedural pain:
- Profile of Mood States (POMS) questionnaire: Used in two studies to evaluate positive and negative affect related to dental treatment prior to emergency endodontic treatment.
